# Employment of Position in Controlling Hæmorrhage

**Published:** 1863-12

**Authors:** Francis B. Quinlan

**Affiliations:** Trin. Coll., Dublin


### EMPLOYMENT OF POSITION IN CONTROLLING HÆMORRHAGE.

By FRANCIS B. QUINLAN, M.D., Trin. Coll., Dublin.

Pain, shock to the nervous system, and hæmorrhage may be fairly considered the principal sources of immediate difficulty and danger in the actual performanceof extensive surgical operations; and as the all but universal employment of anæsthetic agents has, to some degree, neutralized the first two impediments, it may be of advantage to recur to a plan of diminishing venous hæmorrhage, which employed and described in the year 1845, has since been frequently resorted to, although not always with due acknowledgment to Dr. O'Farrell of St. Vincent's Hospital, the distinguished surgeon by whom this plan was first devised. It will be admitted that, while most cases of arterial hæmorrhage are susceptible of comparatively easy control, there is scarcely any bleeding so rapid, so tremendous, or so alarming in its effects as that experienced in the removal of large scrotal tumors, when the enormous tortuous veins, usually found in connection with these growths, have been divided while in a state of repletion; and it is to guard against such hæmorrhage that the plan to which I have alluded is especially directed.

The accuracy of these statements will be easily established by a brief review of some operations of the kind which have been performed with and without having recourse to this plan.

In the first of these cases, a large scrotal tumor, weighing about fifty pounds, was removed by the late Mr. Liston, the veins being in an engorged condition. Upon the first incisions being made, the blood flooded out, to use the words of that celebrated operator, "as from a shower-bath;" the patient rolled in exhaustion and agony from the table, and the operation was completed upon the floor; the patient collapsed, and was with difficulty restored by the energetic exhibition of stimulants. In Mr. Ashton Key's operation, performed upon the Chinese Hoo-Loo, the results were similar, but from the feeble Asiatic temperament of the patient, more disastrous. The operation lasted an hour and three-quarters, and the patient, who had shown some signs of syncope during its continuance, died immediately after its conclusion. It may be observed that in both these cases the genital organs were necessarily sacrificed in an effort to hurry the operation to a conclusion, in order to save the patient from impending death from hæmorrhage.

Results of this character, occurring in the hands of some of the first operators of the day, were sufficiently appaling; and it speedily became evident that, unless some means could be devised to diminish this excessive hæmorrhage, the removal of such tumors must, like the extirpation of bronchocele, be for the present abandoned. It was, therefore, with peculiar satisfaction that the profession learned, in the *Dublin Hospital Gazette*, of February, 1845, that a method of operation had been devised by Dr. O'Farrell, by means of which he had removed an enormous scrotal tumor (fully equal to those removed be Liston and Ashton Key) without difficulty, in eight minutes, and with the loss of only five ounces of blood; the genital organs being preserved, and the patient having made a good recovery, notwithstanding attacks of erysipelas, and various other unfavorable circumstances. Such an announcement could not fail to be in the highest degree gratifying; and it became all the more so when it was found that the importance of Dr. O'Farrell's plan of operation was only equalled by its extreme simplicity. Observing the great change produced in turgid varicose veins of the leg by placing the patient upon his back and elevating the limb, and the immediate arrest of hæmorrhage from such veins which ensues upon the adoption of this position, it occurred to Dr. O'Farrell that, if the enlarged scrotum were held up, a similar withdrawal of the vital fluid would take place, particularly as regards the enlarged and tortuous veins which were the principal sources of hæmorrhage.

The result completely justified the accuracy of this expectation—the more so as the hæmorrhage in these cases had been always observed to be principally of a venous character; the arterial hæmorrhage, in Ashton Key's case, being estimated to be scarcely one-twentieth of the whole.

Since the publication of Dr. O'Farrell's plan, a complete change had occurred in these operations, which have since been performed in rather considerable number, and with an ease and success more or less resembling that experienced in his case. I now recur to the plan, because in two instances of operation, published during the present year, (in one of which an Asiatic was the subject,) it apears to me that the able and successful operators, although adopting the method, omitted, in their reports of the cases, to make due acknowledgment to the author; contrasting, in this respect, with Mr. South, who, in his splendid work on Surgery, gives due prominence to Dr. O'Farrell's plan.

The application of this method is by no means limited to the removal of large scrotal tumors. On the contrary, it has been resorted to by Dr. O'Farrell in cases of considerable innocent tumors of a vascular character; and in amputations he has obtained great advantages by loosely applying the tourniquet, elevating the limb, emptying it of venous blood by manipulation, and then tightening the tourniquet. The limb can thus be kept in a state of comparative anæmia while the amputation is being accomplished; and a loss of blood can be prevented, which, by deteriorating the general quality of the vital fluid, might lay the foundation of much subsequent disease. In fact, the value of a position by which the entrance of arterial blood into a limb will be retarded, and the exit of venous blood facilitated, is almost as useful in the performance of an operation as in the treatment of inflammation.—*London Medical Times and Gazette.*

